# Dealing with disjunct populations of vascular plants: implications for assessing the effect of climate change

**DOI:** 10.1007/s00442-023-05323-y

**Published:** 2023-02-04

**Authors:** Lucia Varaldo, Maria Guerrina, Davide Dagnino, Luigi Minuto, Gabriele Casazza

**Affiliations:** Università di Genova, Dipartimento di Scienze della terra, Ambiente e Vita, Corso Europa 26, I-16132 Genoa, Italy

**Keywords:** Intraspecific variation, Climatic niche, Species distribution model, Northern Mediterranean mountains

## Abstract

Species distribution models are the most widely used tool to predict species distributions for species conservation and assessment of climate change impact. However, they usually do not consider intraspecific ecological variation exhibited by many species. Overlooking the potential differentiation among groups of populations may lead to misplacing any conservation actions. This issue may be particularly relevant in species in which few populations with potential local adaptation occur, as in species with disjunct populations. Here, we used ecological niche modeling to analyze how the projections of current and future climatically suitable areas of 12 plant species can be affected using the whole taxa occurrences compared to occurrences from geographically disjunct populations. Niche analyses suggest that usually the disjunct group of populations selects the climatic conditions as similar as possible to the other according to climate availability. Integrating intraspecific variability only slightly increases models’ ability to predict species occurrences. However, it results in different predictions of the magnitude of range change. In some species, integrating or not integrating intraspecific variability may lead to opposite trend in projected range change. Our results suggest that integrating intraspecific variability does not strongly improve overall models’ accuracy, but it can result in considerably different conclusions about future range change. Consequently, accounting for intraspecific differentiation may enable the detection of potential local adaptations to new climate and so to design targeted conservation strategies.

**Supplementary Information:**

The online version contains supplementary material available at 10.1007/s00442-023-05323-y.

## Introduction

Species distribution models (SDMs) are the most widely used tool to predict species distributions for various aims, including species conservation and assessment of climate change impact (Guisan and Zimmerman 2000). Most studies conducted using SDMs assume that all populations of the species would respond to the environment in the same way (Mota-Vargasa and Rojas-Soto 2016; Qiao et al. 2017). Actually, many species exhibit intraspecific ecological variation and to not consider this ecological differentiation may bias predictions obtained with models (D’Amen et al. 2013; Valladares et al. 2014). Consequently, SDMs at the species level may overlook any difference in relationship between groups of populations and climate and they may result in lower model sensitivity (i.e., lower ability to predict presences), affecting projections of future habitat suitability (Osborne and Suárez-Seoane 2002; Lecocq et al. 2019). Therefore, SDMs at the species level may lead to misplaced conservation plans (Hällfors et al. 2016). This issue may be particularly relevant in species in which few populations with potential local adaptation occur (Lecocq et al. 2019; Pearman et al. 2010). To increase the SDMs’ performance (Gonzalez et al. 2011) and to provide a more robust basis for conservation plans, it is recommended to divide species into subunits with biological significance (Smith et al. 2019).

Defining subunits within a species is a major difficulty in integrating intraspecific niche divergences in SDMs. Ideally, subunits should be defined on the basis of the relationships between regional climate and populations of species (Pearman et al. 2010, Oney et al. 2013, Romero et al. 2014, Valladares et al. 2014). However, this information is almost never available for most species. Consequently, different approaches have been performed to define a priori species subunits, such as: (i) occurrences were spatially portioned into geographic quadrants (Osborne and Suárez-Seoane 2002); (ii) subunits were based on distinct genetic lineages or recognized subspecies (Hernandez et al. 2006; Gonzalez et al. 2011; Oney et al. 2013); or, (iii) they were based on biological differentiation (Lecocq et al. 2019; Marcer et al. 2016). However, few studies have considered disjunct populations as an effective way to integrate intraspecific differentiation into SDMs (but see Hällfors et al. 2016; Chen et al. 2020), although disjunct populations may be frequently locally adapted because of the divergent selection (Fang et al. 2013; Mimura and Aitken 2010; Veatch-Blohm et al. 2017).

Geographical disjunction occurs when individuals from a group of populations cannot interact or can interact very rarely with individuals from other groups because of the distance or physical barriers that prevent interaction (Wells and Richmond 1995). Geographical disjunction by distance may be due to historical (such as past climate change or human intervention) or ecological (such as substrate specificity and long-distance dispersal) factors. Past climate fluctuations may have fragmented previously continuous distributional range causing the extinction of intervening populations and enabling survival only in refugia and/or isolated areas with relictual suitable habitat (Comes and Kadereit 1998; Kropf et al. 2003; Schönswetter et al. 2003). In addition, stochastic long-distance dispersal may have enabled some individuals to reach suitable habitat far from the main distributional range of the species (Kropf et al. 2006; Sanz et al. 2014). Regardless of the causes of disjunction, the low number of immigrants and a possible unequal distribution of the species genetic diversity (Despres et al. 2002) between the disjunct groups result in genetic and demographic disjunctions. Moreover, the geographically distant populations may occur in different biotic contexts (Lozano-Jaramillo et al. 2014; Quiroga et al. 2021), being part of regionally distinct species pools (Gallien et al. 2010; Pellissier et al. 2010) or being exposed to different human pressure (Gehrig-Fasel et al. 2007). These factors might potentially lead to distinct competition regimes, which result in occupying different subset of the inhabitable conditions of the species. The interaction between the different genotypes with the local environments may result in the emergence of ecotypes through adaptations to local conditions (Billings 1973; Leinonen et al. 2009; Keir et al. 2011). These ecotypes are maintained because of the absence or the low level of gene flow (Kawecki and Ebert 2004; Tigano and Friesen 2016). Locally adapted genotypes are expected to have a higher relative fitness in their local habitat than genotypes from other habitats. Some locally adapted populations may become maladapted to new climates because of global warming, while others may be well adapted assuring species survival (Aitken and Whitlock 2013).

In this study, we analyzed how the projections of current and future climatically suitable areas can be affected using SDMs based on the whole species occurrences compared to occurrences’ groups based on separate distribution ranges. We used twelve species with geographically disjunct populations distributed in the Southern European mountains, between the Pyrenees and the South-west Alps. In particular, we were asking the following questions: (1) Do disjunct populations experience different climatic conditions? (2) Do SDMs projections based on geographically disjunct populations differ from projections based on the whole species?

## Materials and methods

### Studied species, occurrence data and climatic layers

We selected 12 plant species characterized by a group of populations that is clearly geographically disjunct from the main range of the species. The distance between main group and disjunct populations ranges from 30 to 500 km (Table 1). Six species have a group of populations in the Alps and the other one in the Pyrenees (having the longest distance between the two groups), one species has a group of populations in the Alps and the other one in Corse. In these seven species, the large geographical distance between populations suggests a very reduced gene flow between groups. Differently, three species have groups of disjunct populations within Alps and two species between Alps and Apennines. In these cases, the distance between groups is shorter and a certain degree of gene flow might still occur. The two disjunct groups of populations were named “core populations” (the larger group) and “disjunct populations” (the smaller group) on the basis of the number of occurrences, without any inference about the genetic or biogeographic relationships between them. To the best of our knowledge, information about possible local adaptations is currently available for none of these studied species.

**Table 1:** Distributional features of the 12 studied species

Species	Core populations		Disjunct populations		Distance between core and disjunct populations (km)
	Number of occurrences	Distribution	Number of occurrences	Distribution	
*Adonis pyrenaica* DC.	41	Pyreneans	24	Southwestern Alps	475
*Allium narcissiflorum* Vill.	542	Southwestern Alps	27	Western Alps	70
*Crocus ligusticus* Mariotti	170	Southwestern Alps	22	Northern Apennines	65
*Cytisus ardoinii* E. Fourn.	98	Southwestern Alps	18	Southwestern Alps	30
*Erysimum collisparsum* Jord.	249	Southwestern Alps	26	Pyreneans	250
*Eryngium spinalba* Vill.	369	Southwestern Alps	43	Southwestern Alps	85
*Gentiana alpina* Vill.	387	Pyreneans	139	Alps	400
*Potentilla nivalis* Lapeyr.	476	Pyreneans	124	Southwestern Alps	360
*Primula hirsuta* All.	377	Pyreneans	252	Alps	500
*Thymelaea dioica* (Gouan) All.	343	Pyreneans	124	Southwestern Alps	400
*Valeriana rotundifolia* Vill.	286	Southwestern Alps	112	Corse	200
*Valeriana saxatilis* L.	104	Eastern Alps	20	Apennines	225

Core populations (the larger group) and disjunct populations (the smaller group) are defined on the basis of the number of occurrences

Occurrence data were obtained from both global and regional databases: Système d’Information et de Localisation des Espèces Natives et Envahissantes (SILENE—www.silene.eu); Sistema de información sobre las plantas de España (Anthos—www.anthos.es); Conservatoire Botanique de Corse (CBNC—http://cbnc.oec.fr); Osservatorio Ligure Biodiversità (Li.Bi.Oss.—ARPAL, Regione Liguria, Italy); and Wikiplantbase #Toscana (http://bot.biologia.unipi.it/wpb/toscana/index.html). For each species, occurrences were spatially filtered retaining randomly only one occurrence per grid cell of about 1 × 1 km. A final data set of 4373 occurrences, ranging from 65 to 629 occurrences per species (Table 1), was used in the analyses.

From the WorldClim data set v.1.4 website (http://www.worldclim.org), we downloaded 19 bioclimatic variables representative of historic (1960–1990) climatic conditions at 1 × 1 km spatial resolution (Hijmans et al. 2005). Furthermore, we downloaded bioclimatic variables for two Representative Concentration Pathways (RCPs), representing moderate and extreme possible future emission trajectories and coded according to a possible range of radiative forcing values in the year 2100 relative to preindustrial values (+2.6 and +8.5 W/m^2^, respectively; IPCC 2014). We used RCPs projections from four general circulation models (GCMs), which represent physical processes in the atmosphere, ocean, cryosphere, and land surface: IPSL-CM5A-LR, provided by Institut Pierre-Simon Laplace; MPI-ESM-LR, provided by Max Planck Institute for Meteorology; HadGEM2-ES, provided by Met Office Unified Model; and CCMS4, provided by Community Earth System Model. Following the approach of Hamann et al. (2015) and Maiorano et al. (2012), we used the first two axes of a principal component analysis (PCA) as environmental variables for species distribution modeling, harmonized on both current and future climates to reduce the transferability issue (Petitpierre et al. 2017). First, we pooled together all the bioclimatic variables for both current and each future scenario (i.e., all the combination of RCPs and GCMs); then, we selected the first two axes of the PCA and re-separated the scenarios. The PCA (see results in Online Resource Table S1) was carried out in R (R Core Team 2019) using the packages ‘ade4’ (Dray and Dufour 2007).

### Niche analysis in environmental space

To test any differentiation in ecological niche in the environmental space between core and disjunct populations, we performed niche analysis in a multivariate space defined by the climatic conditions in which they occur, following the approach of Broennimann et al. (2012). First, for each couple of populations, we calculated the niche overlap using Schoener’s D index (Schoener 1970), which ranges from 0 (no overlap) to 1 (full overlap). This metric is based on the density of species occurrences along the environmental axes of a multivariate analysis (Broennimann et al. 2012) and it is considered one of the best niche overlap metrics (Rödder and Engler 2011). Finally, we used the similarity test to assess whether the observed overlap between the niches of the two groups is significantly higher or lower than expected at random from the backgrounds where the species occur (Warren et al. 2008; Broennimann et al. 2012). In short, the observed niche overlap between the two groups was compared with the overlap measured between the niche of one group and the niche obtained by randomly sampling occurrence points in the background area of the other group. This randomization was repeated 100 times. Significant results indicate that the ecological niches of species are either more or less similar than expected by chance. The similarity test indicates whether the observed niche differentiation is because of an actual selection of different habitats or simply an artifact due to habitat availability in the background areas (Warren et al. 2008). To test whether our results are robust to different choices of background, we defined three background areas using a 5, 10 and 15 km buffer zone around the occurrences of both core and disjunct populations. Both D overlap and similarity test were calculated in R (R Core Team 2019) using the “ecospat” package (Broennimann et al. 2016).

### Species distribution modeling

Species distribution modeling was carried out in R (R Core Team 2019) using the Maxent algorithm (Phillips et al. 2004, 2006) as implemented in the ‘Biomod2’ package (Thuiller et al. 2016). We selected 10,000 random points as pseudo-absence data and a split-sample cross-validation was repeated 10 times, using a random subset (30%) of the initial data set. Model performance was evaluated using both the area under the relative operating characteristic curve (AUC—Hanley and McNeil 1982) and the true skill statistic (TSS—Allouche et al. 2006).

The suitability maps from model projections were converted into binary distribution maps using three different thresholds implemented in the ‘PresenceAbsence’ package (Freeman and Moisen 2008): sensitivity equals specificity (Sens = Spec), maximizing the sum of sensitivity and specificity (MaxSens + Spec), and minimizing the distance between the relative operating curve plot and the upper left corner of the unit square (MinROCdist). These thresholds outperform other commonly used thresholds (Cao et al. 2013; Liu et al. 2005).

We constructed SDMs of both the overall species (hereafter “species model”) and each group of populations (hereafter “core model” and “disjunct model”) over the entire distributional range of the species. In addition, following the approach of Pearman et al. (2010), we considered the area that was predicted to have suitable climatic conditions in one or both groups of populations as an “aggregate” model for the distribution of the species. To obtain a relative score of “goodness” of the aggregate model, we calculated the mean AUC and TSS values for core and disjunct models, according to Gonzalez et al. (2011). In addition, for each studied species, we calculated the sensitivity of all types of models as the proportion of occupied sites that are correctly predicted as suitable by the model under current climatic conditions (Pearman et al. 2010). For SDMs under future climates, we performed an ensemble combining all projections and species were considered occurring in a cell if at least 50% of models projected its occurrence there (i.e., a majority consensus rule).

### Range analysis under future climate

To assess the impact of climate change on the potential distribution of each species, we calculated the percentage of overall range change (RC). This index was calculated separately for each type of model using the following formula: RC = 100 × (RG − RL)/CPR, according to Casazza et al. (2014). RG (range gain) is the number of grid cells not suitable under current condition but suitable under future climate; RL (range loss) is the number of grid cells suitable under current climate but unsuitable under future climate; CPR (current potential range) is the number of grid cells suitable under current climate.

## Results

### Niche analysis

**Fig. 1:**
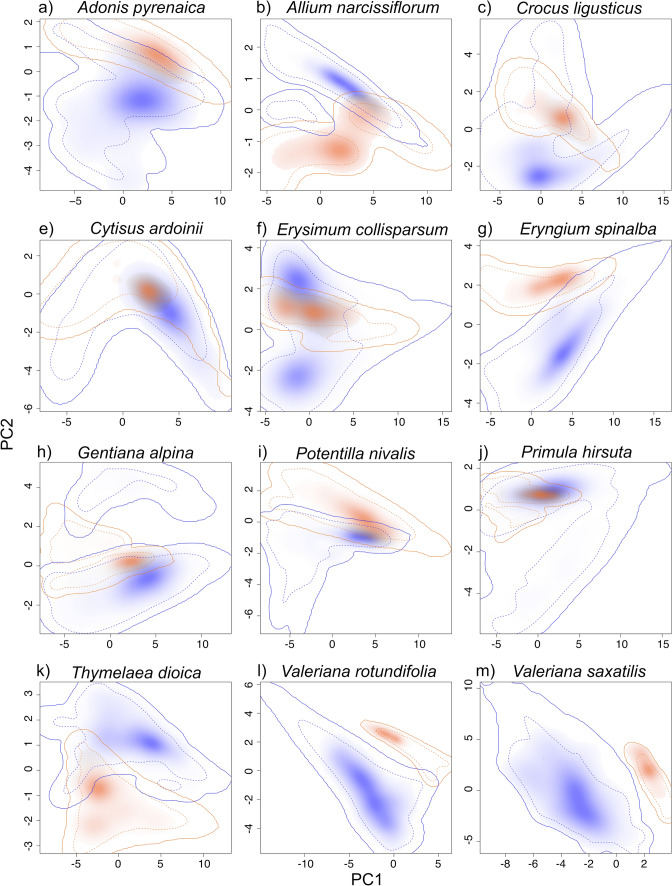
Niches of the core (blue) and disjunct (orange) populations of the 12 studied species. The solid and dashed lines represent 100 and 50% of the entire available environmental space (considering a background area of 10 km around occurrences), respectively. Color shadings illustrate the density of the occurrences of core and disjunct populations in each climatic cell

The niche overlap between the two groups of populations was low (Fig. 1). It ranged from 0 to 0.39 (Table 2, Online Resource Table S2) and was close to 0 in 5 out of 12 species (Table 2, Online Resource Table S2). The similarity test indicated that in 7 out of 12 species, the ecological niche of at least 1 group of populations was significantly more similar to the niche of the other one than expected by considering the differences in the surrounding environmental conditions (Table 2, Online Resource Table S2). Taken together, our results show that in these seven species, the overlap between the two groups is low, but the two groups of populations occupy environments that are significantly more similar to each other than expected by chance.

**Table 2:** Results of niche overlap and niche similarity test between core and disjunct populations

Species	Niche overlap	Similarity test	
		Core vs disjunct	Disjunct vs core
		10 km background	10 km background
*Adonis pyrenaica*	0.14	Ns	More
*Allium narcissiflorum*	0.16	More	Ns
*Crocus ligusticus*	0.30	More	Ns
*Cytisus ardoinii*	0.16	Ns	More
*Erysimum collisparsum*	0.08	Ns	Ns
*Eryngium spinalba*	0.06	Ns	Ns
*Gentiana alpina*	0.27	Ns	More
*Potentilla nivalis*	0.19	Ns	Ns
*Primula hirsuta*	0.39	More	More
*Thymelaea dioica*	0.06	More	More
*Valeriana rotundifolia*	0.00	Ns	Ns
*Valeriana saxatilis*	0.00	Ns	Ns

Background is defined by applying 10 km buffer zones around the occurrence points. Significant results are indicated by ‘less’ for significant divergence or ‘more’ for significant similarity between test and comparison taxa, ‘ns’ indicates not significant results

### Model performance

With few exceptions, AUC and TSS indicated good to excellent performance under current climates for species, core and disjunct models (Table 3). In most cases, evaluation of core and disjunct models was slightly higher than their respective species models. Consequently, the performance of aggregate models (obtained averaging the AUC and TSS values of core and disjunct models) was equal to or higher than species models (Table 3). The sensitivity scores were high in all types of models, but in almost all species, the aggregate models outperformed the species models, better predicting the known species’ occurrences (Table 3).

**Table 3:** Model performance evaluation

Species	Model	AUC (sd)	TSS (sd)	Sensitivity (%)
*Adonis pyrenaica*	Core	0.97 (0.02)	0.86 (0.07)	92.68
	Disjunct	0.99 (0.01)	0.96 (0.03)	95.83
	Species	0.97 (0.01)	0.85 (0.05)	90.77
	Aggregate	0.98 (0.02)	0.91 (0.05)	93.85
*Allium narcissiflorum*	Core	0.94 (0.01)	0.77 (0.02)	89.30
	Disjunct	0.92 (0.03)	0.76 (0.07)	81.48
	Species	0.93 (0.01)	0.74 (0.02)	88.75
	Aggregate	0.93 (0.02)	0.77 (0.05)	91.56
*Crocus ligusticus*	Core	0.98 (0.00)	0.89 (0.02)	95.29
	Disjunct	0.99 (0.00)	0.96 (0.02)	100.00
	Species	0.98 (0.01)	0.89 (0.04)	95.83
	Aggregate	0.99 (0.00)	0.93 (0.02)	95.83
*Cytisus ardoinii*	Core	0.99 (0.02)	0.99 (0.00)	96.94
	Disjunct	0.98 (0.00)	0.91 (0.02)	100.00
	Species	0.99 (0.00)	0.96 (0.01)	94.83
	Aggregate	0.99 (0.00)	0.95 (0.01)	97.41
*Erysimum collisparsum*	Core	0.93 (0.01)	0.73 (0.03)	87.95
	Disjunct	0.94 (0.02)	0.85 (0.04)	92.31
	Species	0.92 (0.01)	0.72 (0.03)	89.82
	Aggregate	0.94 (0.02)	0.79 (0.04)	91.27
*Eryngium spinalba*	Core	0.93 (0.01)	0.76 (0.04)	88.35
	Disjunct	1.00 (0.00)	0.97 (0.03)	97.67
	Species	0.92 (0.01)	0.74 (0.02)	88.83
	Aggregate	0.97 (0.01)	0.87 (0.03)	90.53
*Gentiana alpina*	Core	0.98 (0.00)	0.86 (0.02)	93.02
	Disjunct	0.98 (0.01)	0.88 (0.02)	92.81
	Species	0.96 (0.01)	0.83 (0.02)	90.68
	Aggregate	0.98 (0.01)	0.87 (0.02)	93.73
*Potentilla nivalis*	Core	0.97 (0.00)	0.87 (0.01)	94.75
	Disjunct	0.98 (0.00)	0.89 (0.02)	95.16
	Species	0.97 (0.00)	0.84 (0.01)	92.83
	Aggregate	0.98 (0.00)	0.88 (0.02)	95.33
*Primula hirsuta*	Core	0.97 (0.01)	0.84 (0.02)	92.31
	Disjunct	0.97 (0.02)	0.91 (0.03)	95.24
	Species	0.96 (0.01)	0.83 (0.02)	91.73
	Aggregate	0.97 (0.02)	0.88 (0.03)	93.8
*Thymelaea dioica*	Core	0.93 (0.01)	0.73 (0.02)	84.55
	Disjunct	0.91 (0.02)	0.73 (0.07)	86.29
	Species	0.90 (0.01)	0.66 (0.02)	86.51
	Aggregate	0.92 (0.02)	0.73 (0.05)	90.15
*Valeriana rotundifolia*	Core	0.86 (0.02)	0.58 (0.04)	83.92
	Disjunct	0.92 (0.13)	0.83 (0.25)	99.11
	Species	0.89 (0.01)	0.63 (0.04)	84.42
	Aggregate	0.89 (0.08)	0.71 (0.15)	88.19
*Valeriana saxatilis*	Core	0.89 (0.01)	0.70 (0.03)	86.54
	Disjunct	0.93 (0.04)	0.79 (0.09)	90.00
	Species	0.85 (0.02)	0.61 (0.03)	83.06
	Aggregate	0.91 (0.03)	0.75 (0.06)	89.52

The values of the area under the relative operating characteristic curve (AUC) and true skill statistic (TSS) are the means of the evaluation scores of the 100 runs performed for each type of model. The sensitivity of all types of models is estimated as the proportion of occupied sites that are correctly predicted as suitable by the model under current climatic conditions

### Range analysis under future climate

**Fig. 2:**
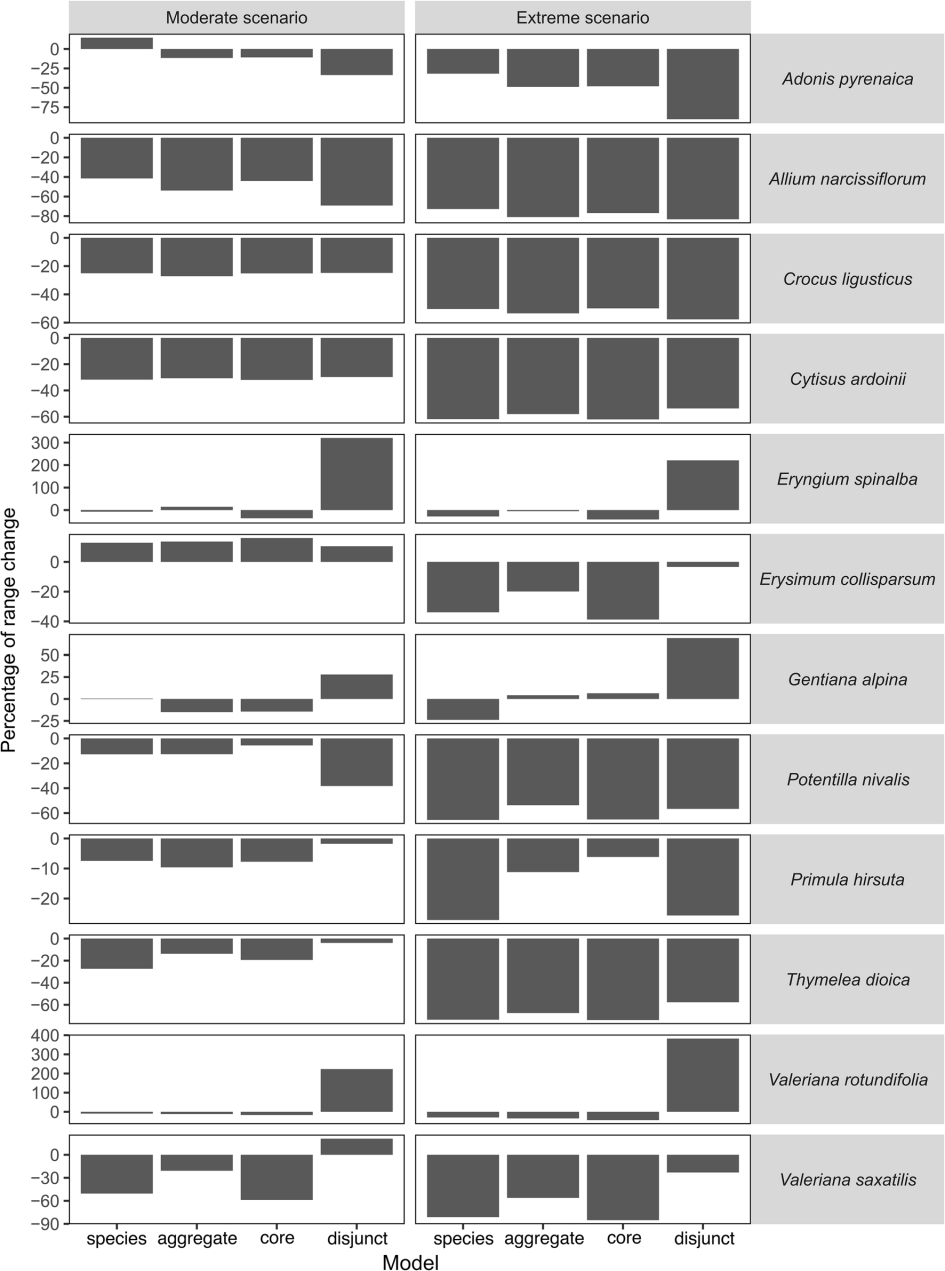
Percentage of range change projected under moderate and extreme future scenarios

**Fig. 3:**
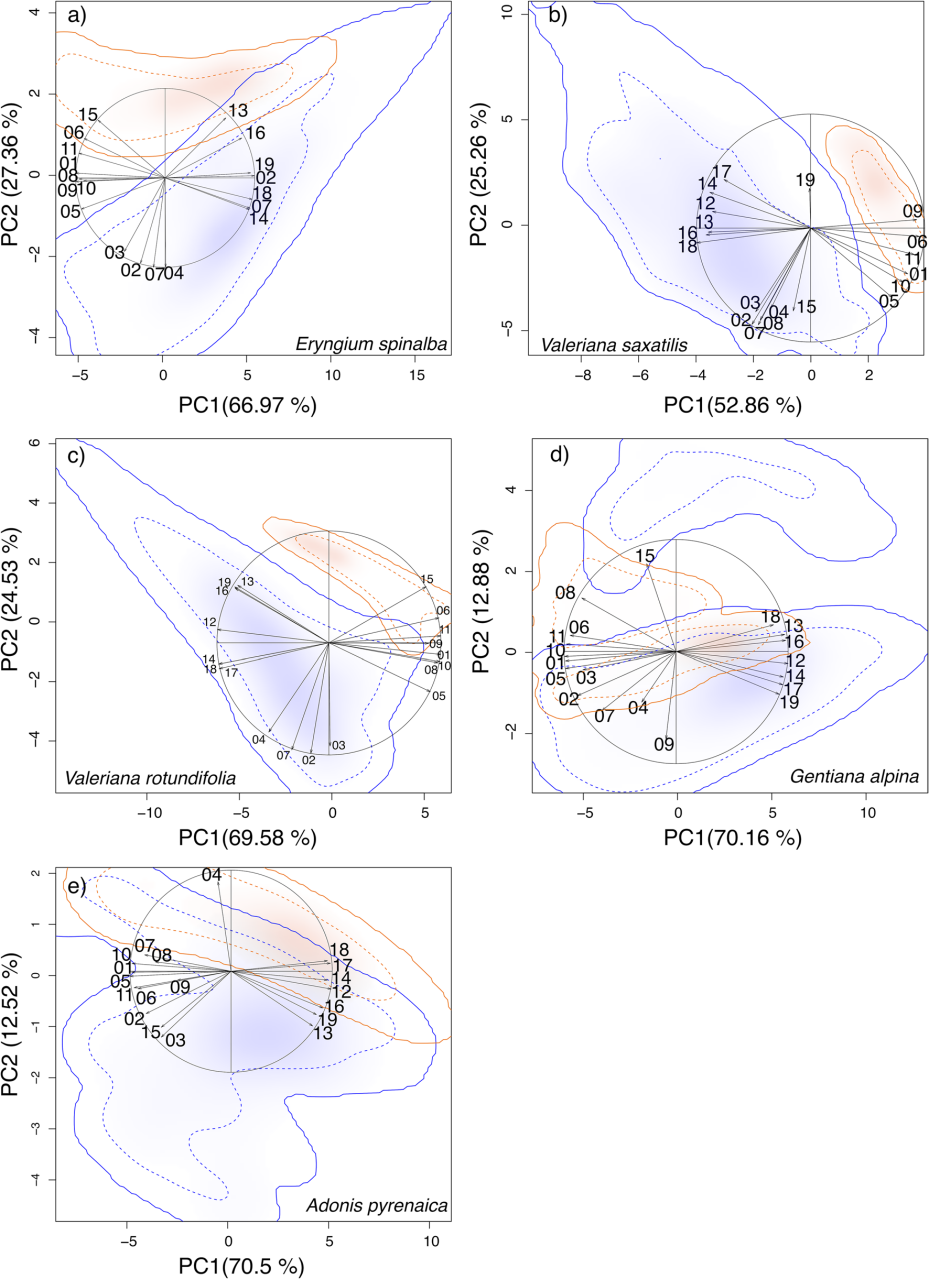
Focus on the niches of the core (blue) and disjunct (orange) populations of five studied species with different trends predicted by SDMs. The correlation plot reporting the contribution of each bioclimatic variable in the niche space to the first and second principal components is shown. The solid and dashed lines represent 100 and 50% of the entire available environmental space (background), respectively. Color shadings illustrate the density of the occurrences of core and disjunct populations in each climatic cell. Bioclimatic variables are: 01 = annual mean temperature; 02 = mean diurnal range; 03 = isothermality; 04 = temperature seasonality; 05 = max temperature of warmest month; 06 = min temperature of coldest month; 07 = temperature annual range; 08 = mean temperature of wettest quarter; 09 = mean temperature of driest quarter; 10 = mean temperature of warmest quarter; 11 = mean temperature of coldest quarter; 12 = annual precipitation; 13 = precipitation of wettest month; 14 = precipitation of driest month; 15 = precipitation seasonality; BIO16 = precipitation of wettest quarter; 17 = precipitation of driest quarter; 18 = precipitation of warmest quarter; 19 = precipitation of coldest quarter

In most species, an overall range contraction (i.e., negative range change) was forecasted under both the moderate and the extreme scenarios, but some differences among model types were detected (Fig. 2). In general, all models projected the same trend in range change and species models projected a higher range contraction than aggregate models. However, despite both species and aggregate models had the same trend, in three cases, the disjunct model projected a range gain, while the core model projected a range loss (i.e., *Valeriana rotundifolia* in both scenarios, *Valeriana saxatilis* in moderate scenario and *Eryngium spinalba* in extreme scenario). Moreover, in four cases, species and aggregate model predicted opposite trends: in two cases (*Eryngium spinalba* and *Gentiana alpina* both under moderate scenario) contrasting range change trends occur also between the core and the disjunct models, while in the other two cases (*Adonis pyrenaica* under moderate scenario and *Gentiana alpina* under extreme scenario), the core and the disjunct models predicted a concordant range change trend. In *Eryngium spinalba, Valeriana saxatilis* and *Valeriana rotundifolia* the niche overlap was very low (0.00–0.06), and the disjunct populations occur under Mediterranean climatic conditions with low values of temperature seasonality and precipitation concentrated during wet period, while core populations occur under temperate (mountain) climate with high values of temperature seasonality (Fig. 3a–c). In *Adonis pyrenaica* and *Gentiana alpina* niche, overlap was higher (0.14 and 0.27, respectively), and the disjunct populations grow under a subset of marginal conditions of core populations having different optimal conditions (Fig. 3d, e).

## Discussion

In this study, we assessed the importance of considering geographically separated populations to predict potential effects of future climate change using SDMs. In fact, these disjunct populations may respond differently to climate change because they may host local adaptation or because they may occur in more suitable climatic conditions in the future. Our results underline the importance of incorporating intraspecific variability in SDMs, given that it can provide different conclusions about future range changes.

### Climatic niche differentiation within disjunct populations

Our results suggest that ecological differentiation among disjunct and core populations occurs, although disjunct populations grow under the available climatic conditions more similar to those of core populations (Table 2, Online Resource Table S2). The niche similarity is in line with previous studies suggesting that disjunct populations maintain the same climatic niche (e.g., arctic-alpine species—Wasof et al. 2015, Corso-Sardinian species—Piñeiro et al. 2007, species ranging from Pyrenees to Alps—Kropf et al. 2008), particularly when disjunctions result from paleoclimatic changes (Winkworth et al. 2015). In line with this observation, the disjunct populations of *Gentiana alpina*—the only studied species for which phylogeographic studies are available—were attributed to vicariance events (Kropf et al. 2006). The ecological differentiation among disjunct and core populations is irrespective of the distance between the central and disjunct populations. In fact, the low degree of niche overlap between core and disjunct populations (Table 2, Online Resource Table S2) may occur because of differences in environmental availability across their geographic ranges (Murphy and Lovett Doust 2007; Dagnino et al. 2016), historical climate changes, or other non-climatic factors (e.g., dispersal limitation and biotic interactions) that limit the distributional range of species resulting in a climatic disequilibrium between populations (Shipley et al. 2013).

In our study, species occurrences are predicted better by the aggregate than by the species model, as suggested by the slightly highest values of sensitivity and accuracy detected in the aggregate model (Table 3). A higher accuracy in aggregate than in species models was observed in several other studies considering intraspecific variability as formally recognized subspecies (e.g., Gonzalez et al. 2011; Oney et al. 2013), genetic lineages (e.g., Marcer et al. 2016; Ikeda et al. 2017) or a combination of them (e.g., Pearman et al. 2010), underlying the importance of considering intraspecific variation to increase accuracy of predictive models (Smith et al. 2019). In fact, species model may underestimate the overall niche of a species having disjunct distribution, resulting in an under-prediction bias for the less widespread group of populations (Pearman et al. 2010; Oney et al. 2013). This may occur when one group of populations occupies a narrower range of climatic conditions than the other group, as observed in most of the studied species (Fig. 1). Conversely, the aggregate model is the sum of the independent core and disjunct models and, consequently, it maximizes the sensitivity value also for the group with the narrowest niche, thus reducing the under-prediction bias. Considerable intraspecific variability occurs in plant species growing along environmental gradients in Mediterranean mountains (Pironon et al. 2017; Casazza et al. 2021; Macrì et al. 2021). For this reason, although we detected niche conservatism in disjunct populations, these populations growing under marginal conditions may generate valuable adaptive genetic combinations because of differential selection pressures (Hereford 2009) and, therefore, they might respond in a different way to climate change (Morente-López et al. 2021; Papuga et al. 2018).

### Intraspecific differentiation and future range changes

In general, the high AUC and TSS values suggest that model predictions are highly accurate. In six species (i.e., *Adonis pyrenaica*, *Allium narcissiflorum*, *Crocus ligusticus*, *Cytisus ardoinii*, *Erysimum collisparsum* and *Valeriana saxatilis*) the number of occurrences in the disjunct populations is closed to the number of occurrences expected to affect the reliability of species distribution models (i.e., 25 occurrences; van Proosdij et al. 2016). However, the high-performance values in disjunct models of these species suggest that the occurrences are not biased and that they adequately represent the environmental gradient used by disjunct populations. Our results suggest that the distributional range of most of studied species will be strongly negatively affected by the climate change (Fig. 2). Nevertheless, the aggregate models generally predicted a slightly less severe range change than the species models (Fig. 2). This result is in line with previous studies including intraspecific (i.e., populations or subspecies) or intra-clade (i.e., sister species) niche variability (Pearman et al. 2010, Benito Garzón et al. 2011, Oney et al. 2013, Valladares et al. 2014) in the models. This pattern may be due to the different ecological niche used by the core and disjunct populations under current climate, as previously discussed. In particular, in the aggregate models, the ecological conditions used by the disjunct populations, that use a narrower and different climatic space than core populations, contribute more to the overall niche of the species than in species models. Combining the separate models of core and disjunct populations, the aggregate model may project a broader suitable area into the future climate than the species model (Oney et al. 2013), resulting in a less negative future range change.

However, despite the low niche overlap between core and disjunct populations, we found the same trend (i.e., contraction, expansion, or stability) both in core and disjunct models and, consequently, in species and aggregate models in most of the cases (Fig. 2), as observed in previous studies (Pearman et al. 2010; Hällfors et al. 2016; Maguire et al. 2018). In four cases (i.e., *Eryngium spinalba* in the pessimistic scenario, *Valeriana saxatilis* in the moderate scenario and *Valeriana rotundifolia* in both scenarios), we detected a different trend in core and disjunct models, even if this difference does not result in a different trend between aggregate and species models. The disjunct populations of these species occur under more Mediterranean climatic conditions than core populations (Fig. 3a–c), so they might increase their suitable areas because of climate change. In fact, in the future, species growing under Mediterranean climate, characterized by hot and arid summer and mild to cool winter, will probably lie within the climatic conditions already experienced at least in some periods of the year and, consequently, these species may be less sensitive to climate change (Thuiller et al. 2006; Tielbörger et al. 2014; Dagnino et al 2020). However, this gain in range of disjunct populations will not be large enough to compensate the range loss of core populations growing under temperate conditions, resulting in an overall range loss both in aggregate and species models. Moreover, under the moderate scenario in *Eryngium spinalba* and *Gentiana alpina* contrasting directions of range change occurring between the core and disjunct models result in a different trend between species and aggregate models (Fig. 2). In *Eryngium spinalba*, under the extreme scenario, range gain is very low in disjunct populations (see above). Differently under the moderate scenario, the weak range loss of temperate core populations is counterbalanced by the high range gain of disjunct populations growing under Mediterranean conditions, resulting in an overall gain in the aggregate models. On the contrary, in the species model, the niche was mainly affected by the temperate conditions under which most populations grow, resulting in an overall range loss. In *Gentiana alpina,* the disjunct populations thrive under a subset of conditions which constitute the marginal conditions for the core populations (Fig. 3d). The future climate change will affect in slightly different way the two groups of populations, resulting in a weak gain in the most thermophilous disjunct populations and in a weak loss of distributional range in the core populations (Fig. 3d). These results suggest that in some species, disjunct populations are likely to occur in new conditions that fall within their climatic tolerance. All the above can assure the survival of some lineages that may provide the raw genetic material enabling the species to adapt and/or shift in response to the climatic change (Budd and Pandolfi 2010). In two other cases (i.e., *Adonis pyrenaica* under moderate scenario and *Gentiana alpina* under extreme scenario), although both the core and the disjunct models projected range contraction, the species and the aggregate models projected a contrasting range change (i.e., range contraction in aggregate model and range expansion in species model) (Fig. 2). This may occur when disjunct and core populations share the same suboptimal conditions (Fig. 3d, e). These suboptimal conditions may be recognized as optimal in the species model but not in the populations models, resulting in an opposite trend of range change (Pearman et al. 2010; Valladares et al. 2014). Differently, because the aggregate model is the sum of the potential ranges provided by disjunct and core populations’ models, the range changes detected by the aggregate model are in accordance with those predicted by the last two.

## Conclusion

In conclusion, our results suggest that integrating intraspecific variability does not strongly improve overall accuracy of SDMs based on all species occurrences, but it can result in considerably different conclusions about future range change (Lecocq et al. 2019). However, the response of disjunct groups of populations to climate change largely depends on the difference between the current climate where they grow and the future climate more than on the difference between niches. Consequently, to account for intraspecific differentiation may enable to point out potential resilience units that may act as potential buffer against adverse effects of climate change and accordingly to design targeted conservation strategies (Chen et al. 2020).

## Supplementary Information

Below is the link to the electronic supplementary material.

Supplementary file1 (DOCX 20 KB)

## Data Availability

Occurrences data are owned by public institutions and can be obtained contacting: SILENE—www.silene.eu; Anthos—www.anthos.es; CBNC—http://cbnc.oec.fr); Li.Bi.Oss.—https://www.regione.liguria.it/open-data/item/7256-libioss-specie-animali-suddivise-nei-principali-gruppi-sistematici_7256.html; and Wikiplantbase #Toscana—http://bot.biologia.unipi.it/wpb/toscana/index.html.
