# Supplementary material for: Dealing with disjunct populations of vascular plants: implications for assessing the effect of climate change
Source: Oecologia. 2023 Feb 4;201(2):421–34. doi: 10.1007/s00442-023-05323-y (PMC9945546; doi:10.1007/s00442-023-05323-y)
Supplement: Supplementary file 1 — Supplementary file1 (DOCX 20 KB) [file 442_2023_5323_MOESM1_ESM.docx]

**Supporting information**

**Title:** Dealing with disjunct populations of vascular plants: implications for assessing the effect of climate change

**Authors**: Varaldo Lucia^1*^, Guerrina Maria^1*^, Dagnino Davide^1^, Minuto Luigi^1^, Casazza Gabriele^1^

^1^ Università di Genova, Dipartimento di Scienze della terra, Ambiente e Vita, Corso Europa 26, I-16132 Genova, Italy.

**Table S1. The amount of variance in current and future climates explained by the first (PC1) and the second principal axis (PC2), and the remaining variance not explained by the first two axes (NE).**

| Species | PC1 (%) | PC2 (%) | NE |
| --- | --- | --- | --- |
| *Adonis pyrenaica* | 53.64 | 17.85 | 28.51 |
| *Allium narcissiflorum* | 48.83 | 33.67 | 17.50 |
| *Crocus ligusticus* | 62.34 | 13.24 | 24.42 |
| *Cytisus ardoinii* | 61.50 | 16.94 | 21.56 |
| *Erysimum collisparsum* | 59.34 | 13.28 | 27.38 |
| *Eryngium spinalba* | 64.93 | 10.87 | 24.20 |
| *Gentiana alpina* | 52.93 | 13.17 | 33.90 |
| *Potentilla nivalis* | 57.54 | 14.20 | 28.26 |
| *Primula hirsuta* | 53.69 | 12.82 | 33.49 |
| *Thymelaea dioica* | 56.45 | 15.69 | 27.86 |
| *Valeriana rotundifolia* | 62.17 | 13.92 | 23.91 |
| *Valeriana saxatilis* | 49.76 | 16.55 | 33.69 |

**Table S2. Results of niche overlap and niche similarity test between core and disjunct populations. Backgrounds are defined by applying 5, 10 and 15 km buffer zones around the occurrence points. Significant results are indicated by ‘less’ for significant divergence or ‘more’ for significant similarity between test and comparison taxa.**

| Species | Niche Overlap | Similarity test | | | | | |
| --- | --- | --- | --- | --- | --- | --- | --- |
|  |  | core vs disjunct | | | disjunct vs core | | |
|  |  | background | | | background | | |
|  |  | 5km | 10km | 15km | 5km | 10km | 15km |
| *Adonis pyrenaica* | 0.14 | ns | ns | ns | more | more | more |
| *Allium narcissiflorum* | 0.16 | ns | more | more | ns | ns | ns |
| *Crocus ligusticus* | 0.30 | ns | more | more | more | ns | ns |
| *Cytisus ardoinii* | 0.16 | ns | ns | ns | more | more | ns |
| *Erysimum collisparsum* | 0.08 | ns | ns | ns | ns | ns | ns |
| *Eryngium spinalba* | 0.06 | ns | ns | ns | ns | ns | ns |
| *Gentiana alpina* | 0.27 | ns | ns | ns | more | more | ns |
| *Potentilla nivalis* | 0.19 | ns | ns | more | ns | ns | more |
| *Primula hirsuta* | 0.39 | ns | more | more | more | more | more |
| *Thymelaea dioica* | 0.06 | ns | more | ns | more | more | ns |
| *Valeriana rotundifolia* | 0.00 | ns | ns | ns | ns | ns | ns |
| *Valeriana saxatilis* | 0.00 | ns | ns | ns | ns | ns | ns |
